# Establishment of the chloroplast genetic system in rice during early leaf development and at low temperatures

**DOI:** 10.3389/fpls.2014.00386

**Published:** 2014-08-11

**Authors:** Kensuke Kusumi, Koh Iba

**Affiliations:** Department of Biology, Faculty of Sciences, Kyushu UniversityFukuoka, Japan

**Keywords:** chloroplast transcription, translation, *Oryza sativa*, low temperature, nucleotide metabolism

## Abstract

Chloroplasts are the central nodes of the metabolic network in leaf cells of higher plants, and the conversion of proplastids into chloroplasts is tightly coupled to leaf development. During early leaf development, the structure and function of the chloroplasts differ greatly from those in a mature leaf, suggesting the existence of a stage-specific mechanism regulating chloroplast development during this period. Here, we discuss the identification of the genes affected in low temperature-conditional mutants of rice (*Oryza sativa*). These genes encode factors involved in chloroplast rRNA regulation (*NUS1*), and nucleotide metabolism in mitochondria, chloroplasts, and cytosol (*V_2_*, *V_3_, ST1*). These genes are all preferentially expressed in the early leaf developmental stage P4, and depleting them causes altered chloroplast transcription and translation, and ultimately leaf chlorosis. Therefore, it is suggested that regulation of cellular nucleotide pools and nucleotide metabolism is indispensable for chloroplast development under low temperatures at this stage. This review summarizes the current understanding of these factors and discusses their roles in chloroplast biogenesis.

## INTRODUCTION

Low temperature is a major abiotic constraint to plant growth. In rice, two stages of development are known to be the most sensitive to low temperatures the young seedling stage and the booting stage (Kaneda and Beachell, 1974; Cruz et al., 2013). At the booting stage, pollen sterility caused by low temperatures decreases the final grain yield. At the seedling stage, low temperatures reduce germination and delay leaf emergence and greening. Leaf chlorosis and yellowing are common symptoms when a low temperature prevails during this stage (Cruz et al., 2013), which suggests the low temperature arrests chloroplast development and functioning.

The effect of a low-temperature environment on chloroplast functions has been studied extensively (Berry and Björkman, 1980; Hasanuzzaman et al., 2013). A low temperature causes swelling of the thylakoid lamellae, vesiculation of the thylakoid, and ultimately breakdown of the entire chloroplast. A low temperature also inhibits electron transport and the carbon assimilation apparatus such as the Calvin cycle, ATP synthase, and ribulose 1,5-bisphosphate carboxylase/oxygenase (RuBisCO; Demmig-Adams and Adams, 1992; Asada, 1999; Yamori et al., 2011; Hasanuzzaman et al., 2013). However, these physiological symptoms have been mainly investigated in mature leaves containing functionally established chloroplasts. The molecular mechanisms underlying early chloroplast development under low temperatures have not yet been extensively studied.

*virescent* is a chlorotic mutant of higher plants causing young leaves to have a reduced chlorophyll content, but the chlorophyll levels recover as they grow (Archer and Bonnett, 1987). In contrast with other chlorotic mutants showing lethality such as *albino*, *chlorina*, and *xantha*, the *virescent* mutants are not terminal, and can reach maturity and produce seeds. Certain classes of the *virescent* mutations that have been reported are low-temperature conditional. They develop chlorotic leaves under low temperatures, but not under higher temperatures, suggesting a temporal aberration in a factor governing chloroplast development under low-temperature conditions. During the past decade, numerous genes responsible for *virescent* mutations have been identified in rice, and they have been shown to be involved in the chloroplast genetic system, including transcription, translation, and nucleotide metabolism. Because many of these genes are expressed temporally during early leaf development, they are probably involved in the establishment of the plastid genetic system at this phase under low temperatures. Here, we introduce four factors involved in chloroplast biogenesis under low-temperature conditions (NUS1, GKpm, RNRS1, and RNRL1) that have been identified through genetic and functional analysis of *virescent* mutants of rice (*v*_1_, *v*_2_, *v*_3_, and *st1*).

## TEMPERATURE-SENSITIVE PHASE OF *virescent* MUTANTS DURING EARLY LEAF DEVELOPMENT

**FIGURE 1:**
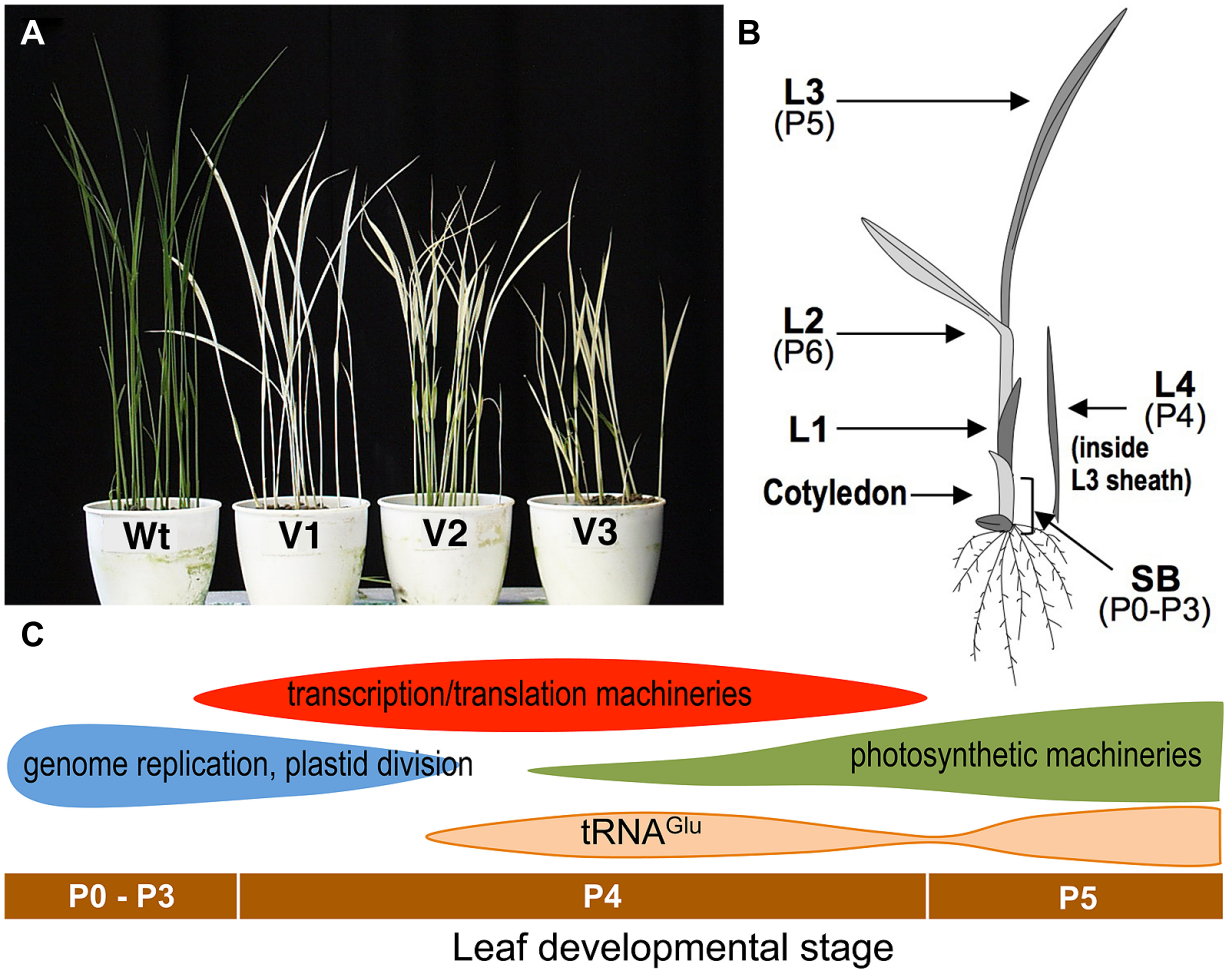
**(A)** Phenotypes of the wild-type (Wt) and *virescent* mutants (*v_1_*, *v_2_*, and *v_3_*) grown at a restrictive temperature (20°C). **(B)** Schematic of a rice seedling with a fully expanded third leaf. L1, L2, L3, and L4 indicate the first, second, third, and fourth leaf, respectively. Developmental stages (P0–P6) are also indicated. shoot base, SB corresponds to a 5 mm section from the bottom of the shoot and contains pre-emerged leaves at P0–P3 stages. **(C)** Plastid gene expression patterns during leaf development (Kusumi et al., 2010a). Horizontal bars indicate the leaf developmental stages.

*virescent-1*, *-2*, and *-3* (*v*_1_, *v*_2_, *v*_3_) were the first *virescent* mutants reported in rice and the mutations have been used as classical genetic markers (Omura et al., 1977). They develop chlorotic leaves at a restrictive low temperature (20°C) but nearly normal green leaves at a permissive higher temperature (30°C; **Figure 1A**). They are often hard to distinguish from each other, showing similar phenotypes. An important characteristic of these *virescent* mutants is that the leaf phenotype is not influenced by growth temperature after its emergence (Iba et al., 1991). This indicates that the leaf phenotype is irreversibly determined by the environmental temperature at a certain developmental stage before emergence. Furthermore, this phenotype can be useful for determining the temperature-sensitive period (TSP), by shifting the temperature from restrictive to permissive during leaf development, or vice versa. This technique was originally performed with *Drosophila* (Suzuki, 1970), in which the TSP for conditional mutants was limited to particular stages of development. Temperature-shift experiments showed that the TSPs of all *v*_1_, *v*_2_, and *v*_3_ mutants was at stage P4 of leaf development (Iba et al., 1991).

Rice has the striking feature of leaf primordia production (plastochron) that is synchronized with leaf emergence (phyllochron) in shoot development (Nemoto and Yamazaki, 1993; Itoh et al., 2005). This regularity of leaf development enables a series of successive stages to be defined, starting with P0 (leaf founder cells), through P1 (youngest primordium), P2, P3, P4, and P5, to P6 (a fully expanded leaf; **Figure 1B**). Anatomical studies have shown that in rice the P4 stage is characterized by rapid leaf blade elongation (Itoh et al., 2005). Leaves at the P4 stage have an initial length of 3–5 mm and reach a final size of about 8–10 cm. Chlorophyll concentration per unit of fresh weight is negligible in the early P4 stage, and increases to about 40% in a mature leaf (Kusumi et al., 2010a). Electron microscopic observations have indicated that chloroplasts in the leaves at the early P4 stage have a small spherical shape (below 1 μm) and poor internal thylakoid structures. After the mid-P4 stage, thylakoid extension and grana formation in chloroplasts has been observed within the mid-portion of the leaf (Kusumi et al., 2010b).

The process of chloroplast development is divided roughly into three steps: (i) plastid division and DNA replication; (ii) establishment of the plastid genetic system; and (iii) activation of the photosynthetic apparatus (Jarvis and Lopez-Juez, 2013; Yagi and Shiina, 2014). These stepwise processes are partially achieved by two plastidial RNA polymerases; a nucleus-encoded phage-type RNA polymerase (NEP), and a plastid-encoded bacterial-type RNA polymerase (PEP; Hajdukiewicz et al., 1997; Yagi and Shiina, 2014). Plastid genes involved in the second step are known to be mainly transcribed by NEP, and those involved in the third step are transcribed by PEP (Yagi and Shiina, 2014). Analyses for chloroplast transcript accumulation revealed that the first step of chloroplast differentiation is likely to start in the leaves at the P0–P3 stages, and will largely finish during the early P4 stage (**Figure 1C**; Kusumi et al., 2010b). The second step occurs significantly in the leaves at mid-P4, and the decline of the second step and onset of the third step take place during late P4 (Kusumi et al., 2010b). The accumulation of tRNA^Glu^, a bifunctional molecule mediating the early steps of chlorophyll synthesis, and the switching of transcription from NEP to PEP (Hanaoka et al., 2005), showed two peaks (late-P4 and P5). The first activation of tRNA^Glu^ can be related to the NEP–PEP transition. Therefore, the TSP of *v*_1_, *v*_2_, and *v*_3_ mutants at the P4 stage suggests that they may be related to the establishment of the chloroplast genetic system, which is the major process occurring at this stage.

## REGULATION OF CELLULAR NUCLEOTIDE POOLS INVOLVED IN THE CHLOROPLAST DEVELOPMENT

**FIGURE 2:**
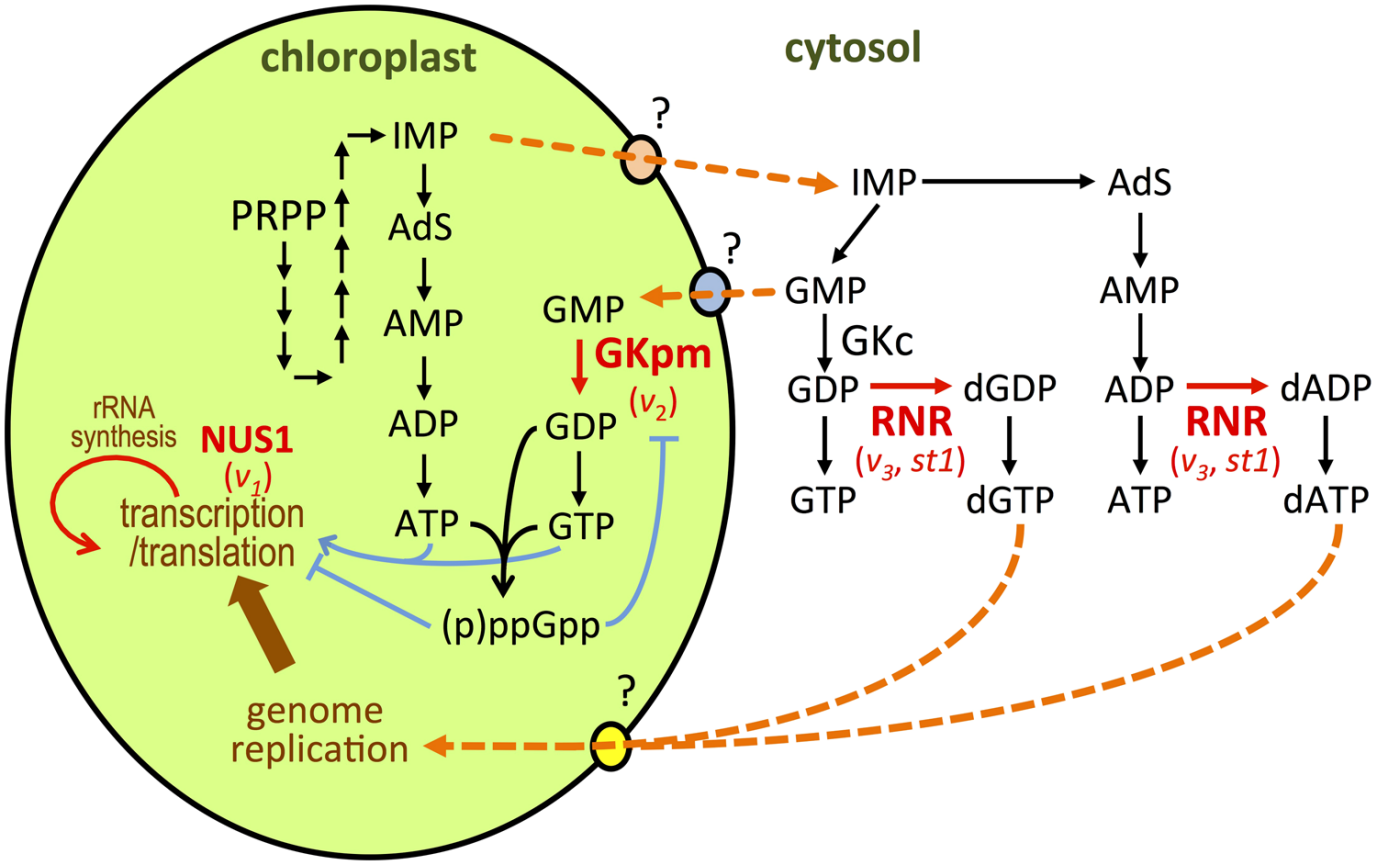
**Representation of *de novo* purine nucleotide synthesis in chloroplasts and cytosol (Sugimoto et al., 2007; Yoo et al., 2009; Kusumi et al., 2011; Nomura et al., 2014).** Proteins whose function has been linked to chloroplast development at cold temperature are depicted in red. The names of the mutation for each protein are indicated below. Orange dotted arrows indicate hypothetical routes for membrane transport or enzyme conversions. PRPP, 5-phosphoribosyl 1-pyrophosphate; IMP, 5’-monophosphate; AdS, adenylosuccinat.

*Virescent-2* (*V_2_*) was the first gene isolated from *virescent* mutants of rice (Sugimoto et al., 2007). Functional analyses showed that *V*_2_ encoded guanylate kinase (GK), a key enzyme in guanine nucleotide biosynthesis that catalyzes the conversion of GMP to GDP (**Figure 2**). In bacterial and animal species, GK is localized in the cytoplasm and participates in maintenance of the guanine nucleotide pools. Plants possess two types of GK; cytosolic GK (GKc) and plastid/mitochondrial GK (GKpm; Sugimoto et al., 2007). Analysis of RNAi knockdown plants showed that GKc is essential for the growth and development of plants, but not for chloroplast development (Sugimoto et al., 2007). *V_2_* is a single-copy gene encoding the GKpm protein. *V_2_-*encoded GKpm predominantly accumulates in developing leaves at the P0–P4 stages (Sugimoto et al., 2007), which is consistent with a temperature-shift experiment in which the *V_2_* gene product was shown to be necessary at the P4 stage. A chloroplast possesses its own nucleoside diphosphate kinase that catalyzes subsequent GDP to GTP conversion (**Figure 2**; Sugimoto et al., 2007; Kihara et al., 2011; Nomura et al., 2014). Therefore, GKpm can limit the GDP/GTP pool in the chloroplast. Reduction of GKpm activity will cause a shortage of the GTP necessary for the assembly and function of the plastid translation machinery. In the *v*_2_ mutant, Val162 has been substituted with Ile, which caused a 20-fold reduction in specific GMP activity (Sugimoto et al., 2007), and severely suppresses chloroplast translation (Sugimoto et al., 2004). Similarly, bacterial GTPases have important roles in ribosome biogenesis and protein translation (Verstraeten et al., 2011). In *Arabidopsis* and tobacco, plastidial GTPases have been reported to be involved in chloroplast rRNA processing and ribosome biogenesis in higher plants (Jeon et al., 2014). It has also been reported that an *Arabidopsis* mutant deficient in GTP-dependent chloroplast elongation factor G developed pale cotyledons and greenish true leaves, as observed in the GKpm-deficient *Arabidopsis* (Albrecht et al., 2006; Sugimoto et al., 2007). This phenotypic similarity suggests the involvement of GKpm in the regulation of plastid translation, via limitation of the GTP pool.

Additionally, it was recently reported that GKpm is a target of regulation by guanosine 3′,5′-bisdiphosphate (ppGpp) in chloroplasts of rice, as well as those of peas and *Arabidopsis* (Nomura et al., 2014). In bacteria, ppGpp is a key regulatory molecule that controls the stringency of responses through direct interaction with protein factors involved in gene expression such as RNA polymerase, translation factors, and DNA primase (Potrykus and Cashel, 2008; Tozawa and Nomura, 2011). In higher plants, ppGpp is synthesized in chloroplasts from GTP (GDP) and ATP (**Figure 2**). Major ppGpp synthase/hydrolase enzymes, named RSH (*RelA/SpoT homolog*), are localized to chloroplasts (Mizusawa et al., 2008). It has also been reported that ppGpp can negatively regulate chloroplast RNA polymerase (Sato et al., 2009) and the elongation cycle of translation (Nomura et al., 2012). This suggests that ppGpp functions as a regulatory molecule in chloroplasts, and interaction between GKpm and ppGpp may limit the GTP (and ATP) pool, which will subsequently retard chloroplast transcription and translation.

Nucleotide biosynthesis in the cytosol is also involved in the regulation of chloroplast differentiation at early leaf development under cold stress. Yoo et al. (2009) showed that the genes responsible for the *v_3_* and *st1* mutants of rice encoded the large and small subunits of ribonucleotide reductase (RNR), RNRL1 and RNRS1, respectively (Yoo et al., 2009). RNR is constructed from large RNR (α) and small RNR (β) subunits, which associate to form an active heterodimer complex (α_2_β_2_) and catalyze conversion of nucleotide diphosphates (NDPs) to deoxyribonucleotide diphosphates (dNDPs; **Figure 2**). Synthesized dNDPs are rapidly converted into dNTPs for DNA replication and repair. Therefore, the RNR activity affects the entire *de novo* nucleotide synthesis pathway *in vivo* (Elledge et al., 1992). As observed in the *v_3_* mutant, *st1* also caused low-temperature-dependent leaf chlorosis. RNRL1 and RNRS1 abundantly accumulated in the leaves at the P0–P4 stages, and this was enhanced by low temperature (Yoo et al., 2009). Both the *v_2_* and *st1* mutations caused missense mutations resulting in reduction of the first ab dimerization, which correlated with the degree of chloroplast disruption (Yoo et al., 2009). This suggests that a threshold level of RNR activity plays an important role in regulating nucleotide flow from the cytosol to chloroplasts. The involvement of cytosolic RNR in plastid nucleotide metabolism is further supported by the report that RNR deficiency causes plastid DNA degradation in pollen in *Arabidopsis* (Tang et al., 2012). Balancing chloroplast biogenesis and cell division during early leaf development would be achieved through optimization of the nucleotide pool in the cellular compartments.

## NUS1 REQUIRED FOR rRNA MATURATION AT LOW TEMPERATURES

In bacteria, synthesis of ribosomes requires a Rho-dependent anti-termination system for the efficient transcription of 16S, 23S, and 5S rRNA from *rrn* operons (Santangelo and Artsimovitch, 2011). All *rrn* operons have anti-terminator sequences in their leader and spacer regions, referred to as BoxB, BoxA, and BoxC, that allow RNA polymerase, modified with protein factors, to transcribe rRNA operons. Previously known protein factors that interact with the anti-terminator include NusA, NusB, NusE, and NusG (Santangelo and Artsimovitch, 2011). Recently, *Virescent-1* (*V_1_*) was identified from a *v_1_* mutant of rice and shown to encode a novel chloroplast RNA binding protein, named NUS1 (Kusumi et al., 2011). The C-terminal region of NUS1 has a structural similarity to the RNA-binding domain of the bacterial NusB, which is classified as alpha helical with seven helices. Accumulation of NUS1 specifically occurs in the developing leaves at the P4 stage, and is enhanced by low-temperature treatment (Kusumi et al., 2011). Although there are no regions identical to bacterial Box regions within the chloroplast *rrn* operon in rice, the gene order of 16S–23S–4.5S–5S and their coding sequences are highly conserved with those of the bacterial *rrn* operon (Bollenbach et al., 2007). RNA-immunoprecipitation and gel mobility shift assays indicated that NUS1 binds to the upstream leader region of the 16S rRNA precursor (Kusumi et al., 2011). The *v_1_* mutant had a nonsense mutation in the helical domain and failed to accumulate the NUS1 protein, and therefore probably represents the null phenotype. In the *v_1_* seedlings grown at low temperatures, the processing and accumulation of chloroplast rRNA and chloroplast translation/transcription was severely suppressed (Kusumi et al., 1997, 2011). Additionally, *Arabidopsis* seedlings deficient in a *NUS1* ortholog also exhibited a similar phenotype (Kusumi et al., 2011). Therefore, NUS1 is likely to be involved in the regulation of rRNA maturation, which occurs at the P4 stage. Bacterial NusB is involved in the protein complex that interacts with RNA polymerase, nascent mRNA, and ribosomes (Santangelo and Artsimovitch, 2011; Bubunenko et al., 2013). Recent proteomics-based techniques have allowed the identification of previously uncharacterized proteins that contribute to the chloroplast genetic system (Majeran et al., 2012; Pfalz and Pfannschmidt, 2013). Majeran et al. (2012) showed that in maize, NUS1 and other factors structurally similar to the bacterial Nus-related factors, such as NusG and Rho, were included in nucleoid-enriched fractions. Examination of the physical interactions among these proteins, and identification of other factors interacting with the NUS1 protein, will be vital for elucidating the role of NUS1 in the regulation of the chloroplast genetic system.

## CONCLUDING REMARKS

Compared with other cereals such as wheat and barley, rice is susceptible to cold stress, probably because of its tropical origin (Cruz et al., 2013). The degrees of low-temperature sensitivity and damage vary according to the growth stage. Yoshida (1981) showed that temperature sensitivity varies between stages and that rice plants have a lower threshold temperature for cold damage during the early young seedling stage (10–13°C) than during the reproductive stage (18–20°C), making them less sensitive to low temperature as young seedlings. In field conditions, sudden low-temperature phases often occur during the early seedling development in spring. Therefore, it is reasonable to infer that rice developed this mechanism to protect leaf and internal chloroplast development against low temperature-induced retardation.

It has been known that processes of chloroplast translation are sensitive to cold stress. Environmental low temperature arrests protein synthesis by causing ribosomal pausing (Grennan and Ort, 2007) or retardation of ribosomal biogenesis and RNA processing (Millerd et al., 1969; Hopkins and Elfman, 1984; Barkan, 1993). Furthermore, loss of translational factors such as ribosomal protein (Rogalski et al., 2008; Fleischmann et al., 2011; Ehrnthaler et al., 2014; Song et al., 2014), translation elongation factor (Liu et al., 2010), rRNA methylase (Tokuhisa et al., 1998) and the RNA binding protein required for RNA processing (Kupsch et al., 2012) leads to sensitivity to low temperatures. These reports suggest the existence of a particular mechanism that protects chloroplast translation against cold stress, which can be expected to be associated with *NUS1*, *V_2_*, *V_3_*, and *ST1*.

The observed involvement of control of translation and nucleotide metabolism in low-temperature tolerance/adaptation has also been reported in bacteria. For example, in *Escherichia coli*, a temperature downshift hampers ribosome function, and ribosomes change their composition to function properly (Akanuma et al., 2012). It has also been reported that cold-shock proteins are often induced not only by low temperatures but also by translational inhibitors, such as chloramphenicol and tetracycline (Weber and Marahiel, 2003). Therefore, a reduction in translational capacity may be interpreted as a cellular signal triggering the cold adaptation response. Furthermore, analyses of bacterial mutants deficient in ppGpp synthesis showed that artificially induced high levels of ppGpp diminish the expression of cold-shock proteins, while low levels increase their production (Potrykus and Cashel, 2008). ppGpp synthesis is triggered by occupation of the ribosomal A-site by an uncharged tRNA (Potrykus and Cashel, 2008). Considering the hampered ribosomal function at low temperature, it is possible that a decrease in cellular ppGpp levels following a temperature downshift plays a physiological role in the regulation of gene expression and adaptation to growth at low temperature. The bacterial NusB protein has also been reported to be involved in cold tolerance. Cells containing a disrupted *nusB* gene are viable under standard growth conditions, but are cold sensitive (Quan et al., 2005). They are defective in rRNA synthesis and have a decreased peptide elongation rate at low temperatures. NusA, another host factor of the Nus complex, is also induced by cold treatment (Phadtare and Severinov, 2010), suggesting the importance of Nus and the anti-termination system in the cold response in bacteria. These similar properties between the chloroplast and the bacterial low-temperature response imply that higher plants have taken over the bacterial protective system in response to low temperature.

Recently, several other genes have been isolated from low temperature-conditional, chloroplast-deficient mutants of rice, such as *OsV4* (*virescent 4*), *wlp1* (*white leaf and panicles 1*), and *tcd9* (*thermo-sensitive chloroplast development 9*; Gong et al., 2014; Jiang et al., 2014; Song et al., 2014). The corresponding genes in *OsV4*, *wlp1*, and *tcd9* mutants encode plastidial pentatricopeptide repeat (PPR) protein, plastid ribosomal protein L13, and a subunit of chaperonin 60 (CP60α) required for chloroplast division, respectively. Similarly to *NUS1*, *V_2_*, *V_3_*_,_ and *ST1*, their functions are speculated to be involved in early chloroplast development at low temperatures (Gong et al., 2014; Jiang et al., 2014; Song et al., 2014). It is possible that these factors are involved in a closely related mechanism to chloroplast protein expression and assembly, which is required at low temperatures, but not essential for chloroplast development during early leaf development at higher temperatures. Therefore, the maintenance of the developing plastid genetic system will be crucial for tolerance of cold at the seedling stage in rice.

## Conflict of Interest Statement

The authors declare that the research was conducted in the absence of any commercial or financial relationships that could be construed as a potential conflict of interest.
